# Dermoscopic Evaluation of Infantile Hemangioma Treated With Topical Timolol

**DOI:** 10.5826/dpc.1004a73

**Published:** 2020-10-26

**Authors:** Rubina Jassi, Sarita Sanke, Ram Chander

**Affiliations:** 1 Department of Dermatology, Lady Hardinge Medical College & Associated Hospitals, New Delhi, India

**Keywords:** hemangioma, infantile hemangioma, timolol, dermoscopy

## Case Presentation

Infantile hemangioma is a common benign vascular proliferation seen in the pediatric age group. Dermoscopy of hemangioma shows lacunae of variable sizes and dilated vessels against a red to reddish blue background [1]. We report the dermoscopic evaluation of regression of infantile hemangioma on topical timolol.

**Figure 1:**
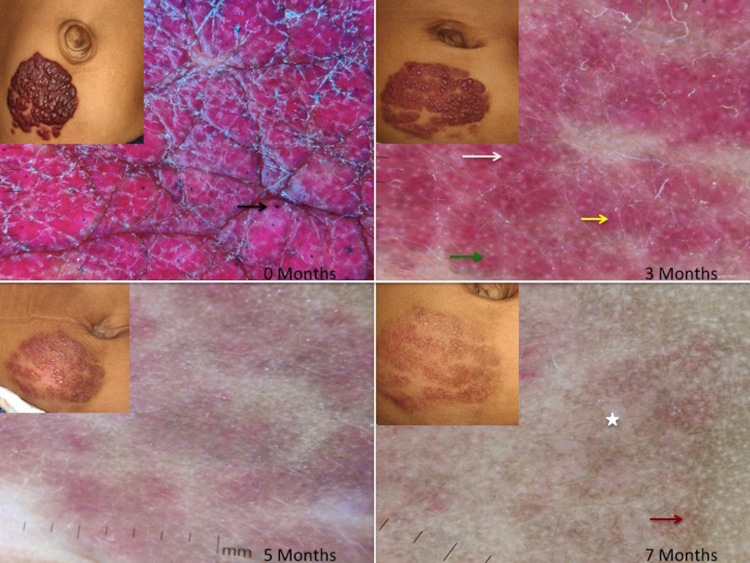
(A) Baseline: diffuse red background with multiple eccrine openings, enhanced skin markings, fine white surface, scaling, and thrombosed capillaries (black arrow). (B) 3-month follow-up: lesion flattened, eccrine openings (white arrow), vascular dots (green arrow), and lacunae (yellow arrow) seen more clearly. Scaling and skin markings disappeared. (C) 5-month follow-up: erythema decreased, lacunae and red dots disappeared, brown pigmentation replacing the erythema. (D) 7-month follow-up: diffuse brown residual pigmentation (red arrow) and empty lacunae (white asterisk).

A 3-month old infant presented with complaints of a red raised proliferative lesion of 5 × 5 cm over the abdomen since 8–10 days of life. A clinical diagnosis of infantile hemangioma was made. The infant was started on topical timolol 0.5% drops twice a day. Dermoscopic evaluation follow-up (DL 3N, polarized, × 20) was done at 0, 1, 3, 5, and 7 months. The erythema was first to respond, followed by decrease in the depth of the lesion. Black dots (black arrow) represent thrombosed capillaries that disappeared in the initial phase of treatment (Figure 1A). White dots (white arrow) represent the eccrine openings that were uninvolved during the entire course of regression (Figure 1B). The classical features, lacunae (yellow arrow) and vascular structures/red dots (green arrow) (Figure 1B), appeared later on at the 3-month follow- up visit once the lesion had flattened, and subsequently lacunae became empty (white asterisk) and the entire erythema was replaced by brown diffuse pigmentation (red arrow) and dots representing residual pigmentation (Figure 1D).

## Teaching Point

Dermoscopic evaluation of hemangioma may help to assess the response of treatment.
